# A Combined Metabolomic and Proteomic Analysis of Gestational Diabetes Mellitus

**DOI:** 10.3390/ijms161226133

**Published:** 2015-12-16

**Authors:** Joanna Hajduk, Agnieszka Klupczynska, Paweł Dereziński, Jan Matysiak, Piotr Kokot, Dorota M. Nowak, Marzena Gajęcka, Ewa Nowak-Markwitz, Zenon J. Kokot

**Affiliations:** 1 Department of Inorganic and Analytical Chemistry, Poznan University of Medical Sciences, 6 Grunwaldzka Street, Poznań 60-780, Poland; jo.hajduk@gmail.com (J.H.); a.klupczynska@gmail.com (A.K.); p.derezinski@gmail.com (P.D.); jmatysiak@ump.edu.pl (J.M.); 2 Obstetrics and Gynecology Ward, District Hospital in Mielec, 22a Żeromskiego Street, Mielec 39-300, Poland; piotrkokot@poczta.onet.pl; 3 Departmentof Genetics and Pharmaceutical Microbiology, Poznan University of Medical Sciences, Święcickiego 4 Street, Poznań 60-781, Poland; dmnowak@ump.edu.pl (D.M.N.); gamar@man.poznan.pl (M.G.); 4 Institute of Human Genetics, Polish Academy of Sciences, 32 Strzeszyńska Street, Poznań 60-479, Poland; 5 Gynecologic Oncology Department, Poznan University of Medical Sciences, Polna 33 Street, Poznań 60-535, Poland; ewamarkwitz@poczta.fm

**Keywords:** gestational diabetes mellitus, mass spectrometry, combined metabolomic and proteomic approach, chemometric analysis

## Abstract

The aim of this pilot study was to apply a novel combined metabolomic and proteomic approach in analysis of gestational diabetes mellitus. The investigation was performed with plasma samples derived from pregnant women with diagnosed gestational diabetes mellitus (*n* = 18) and a matched control group (*n* = 13). The mass spectrometry-based analyses allowed to determine 42 free amino acids and low molecular-weight peptide profiles. Different expressions of several peptides and altered amino acid profiles were observed in the analyzed groups. The combination of proteomic and metabolomic data allowed obtaining the model with a high discriminatory power, where amino acids ethanolamine, l-citrulline, l-asparagine, and peptide ions with *m*/*z* 1488.59; 4111.89 and 2913.15 had the highest contribution to the model. The sensitivity (94.44%) and specificity (84.62%), as well as the total group membership classification value (90.32%) calculated from the *post hoc* classification matrix of a joint model were the highest when compared with a single analysis of either amino acid levels or peptide ion intensities. The obtained results indicated a high potential of integration of proteomic and metabolomics analysis regardless the sample size. This promising approach together with clinical evaluation of the subjects can also be used in the study of other diseases.

## 1. Introduction

Gestational diabetes mellitus (GDM) is a condition with a blood-glucose abnormality that first appears or is first diagnosed during pregnancy. The occurrence of GDM may vary from 1% to 14%, taking into account the population and diagnostic estimation [1,2]. Due to the fact that impaired glucose metabolism may negatively influence the mother’s and infant’s health, the higher risk of complications during the pregnancy, or development of type-2 diabetes after the delivery occurs in women with GDM [3]. Moreover, other adverse effects on the fetus outcome have been well reported [4,5,6].

Therefore, the evaluation of the GDM should be considered at the first prenatal visit [7]. All currently-known diagnostic methods for GDM screening are routinely carried out at 24–28 weeks of gestation and are based on determination of glucose level in the blood [8]. However, the analysis of other endogenous compounds may shed a new light on GDM pathogenesis and provide its earlier detection. Several attempts of GDM characterization at the proteomic level have been already made. By using standard biochemical methods, the following proteins have been estimated as diagnostic biomarkers of GDM: adiponectin [9,10], follistatin-like-3 [11], and sex hormone-binding globulin [12,13,14]. Moreover, applying the mass spectrometry techniques, Kim *et al.* [15] suggested plasma apolipoprotein CIII and apolipoprotein AII as biomarkers. In turn, Liu *et al.* [16] investigating the placenta villi of GDM women, noticed changes in expression of proteins like Annexin A2, Annexin A5, 14-3-3 protein ζ/δ and Ras-related protein Rap1A. Since the amino acids are involved in the regulation of glucagon and insulin secretion, analysis of these metabolites in GDM appears to be well-founded [17,18]. Wang *et al.* [19] stated that the concentrations of five amino acids are highly associated with future development of diabetes mellitus. Furthermore, Cetin *et al.* [20], determining levels of 18 amino acids in maternal and fetal blood samples, concluded that placental transport of amino acid is altered in GDM pregnancies. In maternal blood ornithine was significantly increased, whereas umbilical venous and artery plasma concentrations of 10 amino acids were altered. Those results were not consistent with the previous report of Kalkhoff *et al.* [21], who found elevated levels of six amino acids in plasma samples of patients with GDM.

Although endogenous compounds were tested in GDM women, none of these works proposed a comprehensive determination of amino acids and peptide profiles in the same group of patients. Therefore, the aim of this study was to propose a new approach, which combined the proteomic and metabolomics data and will help in searching for specific GDM biomarkers. Being aware of the small sample size, appropriate multiple statistical analyses were used to obtain the maximum information from the performed study.

## 2. Results and Discussion

The characteristics of women with GDM and the control group are presented in Table 1. Results of the performed tests indicated that there were no statistically significant differences between the study and control groups.

**Table 1:** Characteristics of the study group (GDM patients) and control group (normal pregnancy women). The references indicate the distribution shape of the appropriate variable and univariate statistical test, which was used for comparison of the variable between two groups depending on the distribution shape: * Not normal distribution, Mann–Whitney *U* test; ** Normal distribution, *t*-test.

		Study Group (*n* = 18)	Control Group (*n* = 13)
**Age (years)**	Average	31.5	31.2
	Median	31	31
	Range	20–42	26–35
	*p*-value	0.749 *	
**Body Mass Index BMI (kg/m^2^)**	Average	26.8	24.6
	Median	26.8	24.2
	Range	22.4–32.0	19.5–34.6
	*p*-value	0.074 **	
**Gestational Age at Sampling (Weeks)**	Average	27.1	26.0
	Median	27.5	25
	Range	24–28	24–28
	*p*-value	0.101 *	
**Gestational Age at Delivery (Weeks)**	Average	37.6	38.2
	Median	38	38
	Range	34–40	35–41
	*p*-value	0.347 *	
**Birth Weight (g)**	Average	3281.3	3403.3
	Median	3281	3403
	Range	2200–4100	2390–4480
	*p*-value	0.491 **	
**Pregnancy**	First	8 (44.4%)	5 (38.5%)
	Second	4 (22.2%)	8 (61.5%)
	Third	3 (16.7%)	0
	Fourth	3 (16.7%)	0
**Apgar Score**	10	12 (66.7%)	11 (84.6%)
	9	5 (27.8%)	1 (7.7%)
	8	1 (5.6%)	1 (7.7%)
**GDM in Family**	Yes	2 (11.1%)	2 (15.4%)
	No	16 (88.9%)	11 (84.6%)
**Diabetes Mellitus in Family**	Yes	6 (33.3%)	2 (15.4%)
	No	12 (66.7%)	11 (84.6%)

Free amino acid profiles were obtained using liquid chromatography-electrospray ionization-triple quadrupole tandem mass spectrometry LC-ESI-QqQ-MS/MS. In analyzed samples levels of 34 amino acids were determined and their concentrations are listed in Table S1 in the Supplementary Materials. Eight amino acids (*o*-phospho-l-serine, *o*-phosphoethanolamine, l-homocitrulline, argininosuccinic acid, l-anserine, l-carnosine, cystathionine, l-homocysteine) occurred in the plasma samples below the limit of quantification or were not present in the examined specimens. *t*-tests and Mann–Whitney *U* tests showed statistically significant differences in the levels of four free amino acids: l-asparagine (Asn), l-citrulline (Cit), l-valine (Val), and l-ornithine (Orn) between the analyzed groups (Table 2). Three of the above mentioned amino acid levels were decreased (Asn, Val and Orn), whereas the elevated concentration of Cit was observed in samples from GDM patients in comparison with the control group.

**Table 2:** Peptide ions (*m*/*z*) and amino acids that differ significantly between the study and control group, identified by univariate analysis (*t*-test or Mann–Whitney *U* test).

Compound	*p*-Value
Asn	0.001
2913.15	0.002
4111.89	0.002
3430.44	0.003
1488.59	0.006
4590.58	0.007
3158.72	0.011
Cit	0.015
1370.10	0.026
2168.63	0.026
2210.83	0.027
4437.76	0.028
Val	0.029
1419.58	0.032
Orn	0.038
1435.03	0.048

**Figure 1:**
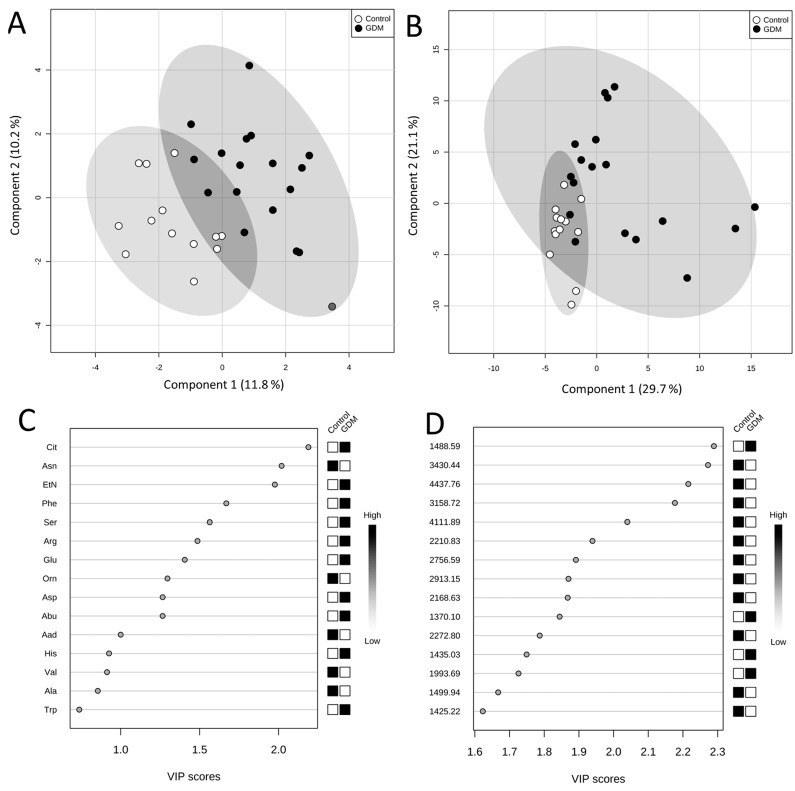
Score plots between first and second latent variable obtained in PLS-DA using plasma free amino acid concentrations (**A**) and peptide ion peak intensities; (**B**) Important variables; (**C**) plasma free amino acids; (**D**) peptide ions identified by PLS-DA.

After application of Partial Least Squares Discriminant Analysis (PLS-DA) a good separation between normal and GDM pregnancies was attained (Figure 1A). The Variable Importance in Projection (VIP) scores rank the amino acids according to their importance in the observed separation between groups (Figure 1C). The following five amino acids were indicated as variables of the highest significance in the model: Cit, Asn, EtN, Phe, and Ser. Due to a propensity for data overfitting, the PLS-DA model was validated using permutation tests. The entire analysis was repeated 1000 times with permutated group labels, and then results with wrong group labels were compared with those of correct group labels. The obtained empirical *p*-value was 0.016, which ensures PLS-DA model’s reliability.

It is noteworthy that two amino acids with the highest VIP score in PLS-DA and two amino acids with the lowest *p*-value obtained in univariate analysis were the same. Hence, the performed statistical analyses showed that Asn and Cit are most related to the occurrence of GDM during pregnancy. Mochida *et al.* [22] found the elevated level of Cit in plasma of rats with type 1 diabetes and this abnormality became more pronounced along with the progression of hyperglycemia. The identified increased levels of urea-cycle metabolites strongly suggest up-regulation of the urea cycle in diabetes mellitus [23]. Additionally, the study conducted by De Luca *et al.* [24] showed that platelet levels of most amino acids, in particular Asn, tended to be significantly lower in patients with type 1 diabetes, suggesting that amino acids changes reflect the altered carbohydrate metabolism and microvascular and macrovascular complications. Our findings are in agreement with these reports and the abovementioned metabolites and metabolic pathways should be further investigated as a promising source of GDM biomarkers.

One of the requirements of Discriminant Function Analysis (DFA) was the number of variables not exceeding the value of six for the given sample size in the presented study. Thus, in order to have six variables, the three most differentiating amino acids, according to univariate analysis (Asn, Cit, Val) and three amino acids with the highest VIP score according to multivariate analysis (Cit, Asn, EtN) were subjected to DFA. The model consisted of four predictors (model 1, Table 3), because two of the amino acids (Asn and Cit) fulfilled both abovementioned criteria. The obtained results showed that the set of four predictors was effective in predicting group membership. The *post hoc* classification matrix was constructed and the sensitivity and specificity values for the model 1 were calculated. The results showed that the occurrence of GDM in pregnant women was predicted with the sensitivity of 94.44% while the absence of the disease was predicted with the specificity of 69.23% with the total group membership classification value of 83.87% (model 1, Table 3). Despite very high sensitivity of the model 1, the calculated specificity was unsatisfactory. Partial Wilks’ Lambda values, which refer to the contribution of the particular predictor to the discriminatory power of the constructed model 1, indicated the significant role of EtN in this model, followed by Cit, Asn, and Val (Table 3). The lower the partial Wilks’ Lambda, the higher the discriminatory power of the respective variable.

**Table 3:** Discriminant function analysis. Comparison of the models constructed based on three sets of predictor variables.

	Model 1		Model 2		Model 3	
	Model Parameters					
**Wilks’ Lambda**	0.4849		0.5777		0.3871	
**F**	6.9045		3.6546		6.3324	
***p*-value**	<0.0006		<0.0128		<0.0004	
**Sensitivity**	94.44%		77.78%		94.44%	
**Specificity**	69.23%		76.92%		84.62%	
**Total Group Membership Classification**	83.87%		77.42%		90.32%	
	**Variables Included to the Model**					
	**Variable**	**Partial Wilks’ Lambda**	**Variable**	**Partial Wilks’ Lambda**	**Variable**	**Partial Wilks’ Lambda**
	Asn	0.9926	1488.59	0.8619	1488.59	0.9607
	EtN	0.9310	2913.15	0.9290	2913.15	0.9966
	Cit	0.9890	3430.44	0.9966	4111.89	0.9846
	Val	0.9975	4111.89	0.9884	Asn	0.9082
			4437.76	0.9999	EtN	0.8845
					Cit	0.9064

Peptide profiles were obtained using matrix-assisted laser desorption/ionization—time of flight mass spectrometry MALDI-TOF-MS. In the analyzed samples, 150 detected peaks of corresponding peptide ions with S/N above 10 and their intensities were subjected to statistical analyses (Table S2). Univariate analyses were performed in order to select peptide ions, which discriminate the study group from the control group. *T*-tests showed statistically significant differences in intensities of twelve peptide ions (Table 2).

In PLS-DA of peptide profiles a clear grouping of GDM and control group was obtained (Figure 1B). The explained variances for the first and second latent variables were higher than those of the PLS-DA of amino acids (29.7% and 21.1% compared to 11.8% and 10.2%, respectively). The performed permutation tests, consisted of 1000 permutations, showed that the separation is statistically significant (*p* = 0.012). Peptide ions of the following *m*/*z* were indicated as the most important variables in the model: 1488.59; 3430.44; 4437.76; 3158.72, and 4111.89 (Figure 1D). The following peaks with discriminatory power: 9122 Da, 9412 Da, 9701 Da and 17,105 Da were proposed by Kim *et al.* [15], in the study that concerned GDM population as well. However, the obtained results were not correlated to our findings mainly due to the different time of sampling and applied analytical methodology.

DFA was performed using three peptide ions with the lowest *p*-value obtained during univariate analyses and three peptide ions with the highest VIP scores as predictors. Since one peptide ion was common, the set of predictors in DFA included the following five *m*/*z* peptide ions: 2913.15; 4111.89; 3430.44; 1488.59; 4437.76 (model 2, Table 3). The obtained results for model 2 indicated its effectiveness in predicting group membership. The highest contribution to the discriminatory power of the constructed model 2 had the peptide ion of *m*/*z* 1488.59. This was indicated by the lowest partial Wilks’ Lambda value (Table 3). The *post hoc* classification matrix allowed to calculate the sensitivity and specificity of the model 2, which were equal to 77.78% and 76.92%, respectively (Table 3). Although the total group membership classification value of 77.42% was lower than in the model 1 constructed using amino acid concentrations, model 2 was characterized by higher specificity compared with model 1.

The statistical analysis of linked proteomic and metabolomic data was performed in order to estimate the utility of combined “omic” approach in the detection of GDM. Variables with the lowest partial Wilks’ Lambda values (three amino acids and three peptide ions) were chosen for a combined DFA as predictors (model 3, Table 3). The obtained results indicated that the set of these six predictors (model 3) was more effective in predicting group membership than models 1 and 2 (Table 3). The combination of proteomic and metabolomic data yielded the model with the best discriminatory power among all constructed models. Partial Wilks’ Lambda values indicated that the highest contribution to the discriminatory power of the model 3 had amino acid EtN followed by Cit, Asn, and peptide ions of *m*/*z* 1488.59; 4111.89, and 2913.15 (Table 3). The sensitivity, specificity, and total group membership classification value calculated from the post-hoc classification matrix of model 3 were the highest among all constructed models (Table 3). Although three amino acids were mainly responsible for classification of model 3, the incorporation of peptide ions to the model led to achieve its higher specificity, which was insufficient for model 1. Thus, the classification of the samples to the study or control group based on the combined peptide ion peak intensities and free amino acids levels was more accurate than the classification based on peptide or amino acid profiles separately.

Additionally, for better description of the combined model, the identification of chosen peptide ions (LP mode *m*/*z* 1488.59; 4111.89 and 2913.15) was performed using liquid chromatography—matrix-assisted laser desorption/ionization—time of flight/time of flight tandem mass spectrometry LC-MALDI-TOF/TOF-MS/MS. The concentrated sample eluent was injected on the column and single compound was spread into 4–8 spots. Subsequently, the MS spectra were evaluated in the reflector mode in the 700 to 3500 Da mass range. Additionally, the mass range of 900 to 4500 Da was checked. The applied methods allow to detect monoisotopic mass of *m*/*z* 1487.6368 and 2911.4712, however lacking the *m*/*z* 4111.89 signal. Non-resolved isotopic peaks in the linear mode of MALDI-TOF-MS profiling were obtained mostly due to the low resolution of the method, caused by spatial and energy spread as well as peak broadening. Contrary to these, in the reflector mode the resolution enables the more sufficient baseline separation of all the isotopic peaks, giving higher mass accuracy. The identification data are enclosed in section Supplementary Materials in Table S3. The MS/MS fragmentation of the 2911.4712 precursor resulted in the identification of the peptide sequence of R.GNTEGLQKSLAELGGHLDQQVEEFRR.R (Figure S1) with significant hit in the Mascot database search to the Apolipoprotein A-IV (APOA4_HUMAN). The protein is known to be involved in lipid metabolism, presenting lipid transporter activity. In the recent study of the visceral adipose tissue in pre-obese patients with type 2 diabetes, an increased level of the apolipoprotein A-IV was reported [25]. Therefore, the changes in the level of this protein could be predictable for diabetes mellitus. Moreover, the apolipoprotein A-IV is showing discriminatory power in our research and can serve as a promising indicator of molecular dysfunction in GDM.

Often, the MS/MS fragmentation is poor when non-tryptic peptides are analyzed and the fragments do not have necessarily a R or K at the C-terminus which favor the protonation and the fragmentation into y-ion series. On that reason the *m*/*z* 1487.6368 might not succeed in any reliable score identification via Mascot database. Additionally, on the MS spectrum the mass 1487.6368 Da of the isotopic cluster may represent the sodium adduct [M + Na]^+^ of the high intense *m*/*z* signal 1465.6566, where the identification resulted with significant hit to Fibrinogen α chain (FIBA_HUMAN) with a peptide sequence of A.DSGEGDFLAEGGGVR.G. The retention time with maximum abundance for these ions is similar. Nevertheless, by comparing the MS/MS spectra of both *m*/*z* signals (*m*/*z* 1487.6368 *vs.* 1465.6566) it is possible to observe the unique non-overlapping fragments and sequence tags are not identically to the one in MS/MS of 1465.6566 (Figure S2). In the MS-BLAST search the peptide sequence AEEAAPSDRMPSAR of *m*/*z* 1487.6368 is showing homology to Kinesin-like protein (KIF1C_HUMAN). Therefore, further investigation should be done to undoubtedly identify *m*/*z* 1487.6368.

## 3. Experimental Section

### 3.1. Chemicals and Reagents

LC-MS grade acetonitrile (ACN), water, ethanol, acetone, and isopropanol, and HPLC-grade methanol were provided by J.T. Baker (Center Valley, PA, USA). The matrix α-cyano-4-hydroxycinnamic acid, Peptide Calibration Standard, and Protein Calibration Standard I, as well as AnchorChip 800 µm were supplied by Bruker Daltonics (Bremen, Germany). Trifluoroacetic acid (TFA) and ammonium acetate were purchased from Sigma-Aldrich (St. Louis, MO, USA). Deionized water was obtained from Millipore Simplicity UV water purification system (Waters Corporation, Milford, MA, USA). Formic acid and heptafluorobutyric acid were purchased from AB Sciex (Framingham, MA, USA).

### 3.2. Subjects and Sample Collection

The project was approved by the Bioethical Commission of Poznan University of Medical Sciences (decision No. 200/13). All patients signed a written consent and fulfilled a detailed survey. The diagnostic criteria of GDM were based on Polish Gynecological Society standards [26]. The initial determination of blood glucose level was performed at the beginning of the pregnancy during the first gynecologist visit, in order to exclude previously-undetected disorders of carbohydrate metabolism. All women were examined for GDM using a 2 h 75 g oral glucose tolerance test between 24 and 28 weeks of gestation. Patients with an abnormal result of plasma glucose level were approved as a study group. Since it has been stated that protein metabolism is normalized in insulin-treated women with GDM, eight insulin-depended cases were excluded from the study and only diet-treated women with GDM were considered in further investigations (*n* = 18) [27]. Moreover, the patients with other additional chronic conditions were rejected. In order to diminish a population variability effect, the pregnant women with normoglycemia and with corresponding age, BMI, ethnicity were recruited as a control group (*n* = 13). All samples were collected in the District Hospital in Mielec, between 24 and 28 weeks of gestation from healthy and GDM pregnant women. Venous blood samples were taken into EDTA tubes and further were centrifuged at 700× *g* for 10 min. The plasma supernatant was aliquoted and stored in −80 °C.

### 3.3. LC-MS/MS Determination of Free Amino Acids

The free amino acids were analyzed in plasma samples using aTRAQ™ kit (AB Sciex, Framingham, MA, USA). The validated method along with sample preparation procedure was described in detail by Matysiak *et al.* [28]. The measurement of amino acid levels was performed with a HPLC instrument 1260 Infinity (Agilent Technologies, Santa Clara, CA, USA) interfaced to a 4000 QTRAP mass spectrometer (AB Sciex, Framingham, MA, USA) with an electrospray ionization (ESI) source. For separation of amino acids an AB Sciex C18 column (5 μm, 4.6 mm × 150 mm) was used with a flow rate of 800 μL/min. The chromatographic separation was carried out using water (solvent A) and methanol (solvent B), both containing 0.1% formic acid and 0.01% heptafluorobutyric acid. A gradient started from 2% to 40% of B from 0 to 6 min, maintained at 40% of B for 4 min, then increased to 90% of B until 11 min and held at 90% of B for 1 min. After 12 min the gradient decreased to 2% of B. From 13 to 18 min the mobile phase composition was unaltered. The injection volume and separation temperature were set at 2 µL and 50 °C, respectively. The ESI source settings were the following: ion spray voltage, 4500 V; source temperature, 600 °C; ion source gas one, 60 psig; ion source gas two, 50 psig and curtain gas, 20 psig. Schedule multiple reaction monitoring mode was applied using nitrogen as a collision gas. The mass spectrometer worked in positive-ionization mode with entrance potential, 10 V; declustering potential, 30 V and collision cell exit potential, 5 V. All the transitions and MS parameters were presented by Matysiak *et al.* [28]. Data acquisition and processing were conducted by the Analyst 1.5 software (AB Sciex, Framingham, MA, USA).

### 3.4. MALDI-TOF-MS Peptide Profiling

Prior MALDI-TOF-MS analyses, all samples were purified and concentrated with the use of C18 pipette tips based on solid phase extraction (ZipTip, Millipore, Bedford, MA, USA). The plasma samples were diluted in 1:5 with 0.1% TFA in water and then loaded onto the C18 tips, passing it in 10 repetitions. For the washing procedure 0.1% TFA in water was used, and peptide fractions were eluted with 50% ACN solution. For the MS measurement, 1 μL of each eluted sample fraction was mixed with 10 μL of α-cyano-4-hydroxycinnamic acid matrix solution (0.3 g/L HCCA in ethanol:aceton 2:1 *v*/*v*), and 1 μL of the mixture sample/matrix was taken and spotted four-fold onto the MALDI AnchorChip 800 μm target (Bruker Daltonics).

For the linear peptide profiling the UltrafleXtreme MALDI–TOF/TOF mass spectrometer (Bruker Daltonics) was used. The instrument settings were as follows: linear positive mode; ion source 1, 25.09 kV; ion source 2, 23.79 kV; lens, 6.40 kV; pulsed ion extraction, 260 ns; matrix suppression mass cut off, *m*/*z* 700 Da. The spectra were recorded in 1000–10,000 Da mass range, accumulating 2000 shots per spectrum and each plasma sample was prepared in four MALDI spots repetition. By routine, the standards calibration mixture (1:5 mixture *v*/*v* of Peptide Calibration Standard and Protein Calibration Standard I) was analyzed for external calibration of the mass spectrometer. The average mass deviation was better than 100 ppm. The spectral data were acquired and automatically saved with FlexControl 3.4 (Bruker Daltonics) and FlexAnalysis 3.4 software (Bruker Daltonics). The preprocessing steps were performed using a MALDIquant 1.10 [29]. Inter-day and intra-day reproducibility of MS spectra obtained after ZipTip fractionation method was evaluated. The mean coefficient of variance (CV) for eleven selected *m*/*z* peaks with relative intensity above 0.5 was less than 10% [30].

### 3.5. Data Processing of MALDI-MS Spectra

The intensities in a single spectrum were transformed to a square root scale for variance stabilization and smoothed using a Savitzky–Golay algorithm. The spectrum background was evaluated using the statistics-sensitive non-linear iterative peak-clipping (SNIP) algorithm and used for baseline correction. The intensities of multiple spectra were normalized using median of intensities. The features with signal-to-noise ratios (S/N) higher than 10 were detected as peaks. The peaks affiliated with the same mass were aligned by the statistical regression-based approach using the identification of landmark peaks and the estimation of a non-linear warping function. Before peak detection, all technical replicates were averaged.

### 3.6. LC-MS/MS Identification of Selected Ion Peptides

The undigested eluent (50% ACN, 0.1% TFA) from the ZipTip preparation of GDM sample was subjected to nano-LC separation. The system consisted of: nanoflow HPLC system EASY-nano LC II (Bruker Daltonics) and fraction collector Proteineer-fc II (Bruker Daltonics). Peptides and small proteins were concentrated on a trap column, NS-MP-10 BioSphere C18 (NanoSeparations: Nieuwkoop, The Netherlands) (5 μm particle size, 120-Å pore size, 100 μm inner diameter, 20 mm length) and separated on a Thermo Scientific Acclaim PepMap 100 column C18 (Thermo Scientific: Sunnyvale, CA, USA) (3 μm, 100 Å, 75 µm × 150 mm) by linear gradient of water (mobile phase A) and 90% ACN (mobile phase B), both containing 0.05% TFA. The gradient elution method was: 2%–50% B in 96 min. The flow rate was maintained at 300 nL/min and the injection volume was 2 µL. In total, 384 fractions of separated sample were mixed with matrix solution and spotted onto an AnchorChip™ MALDI target. 80 nL of eluent was mixed with 420 nL matrix solution per fraction. Matrix solution was created by mixing: 748 μL of 95:5 (*v*/*v*) acetonitrile: 0.1% TFA, 36 μL of saturated solution of HCCA in 90:10 (*v*/*v*) acetonitrile: 0.1% TFA, 8 μL of 10% TFA and 8 μL of 100 mM ammonium phosphate monobasic. The instruments were controlled using HyStar 3.2 software (Bruker Daltonics). Mass spectrometry analysis was performed using the MALDI-TOF/TOF instrument (UltrafleXtreme, Bruker Daltonics) equipped with a SmartBeam II laser (Bruker Daltonics). The instrument setting for MS analysis was as follows: ion source one, 25.09 kV; ion source two, 22.59 kV; lens voltage, 7.89 kV; pulsed ion extraction time, 120 ns; matrix suppression mass cut off, *m*/*z* 700. For the external calibration of the MS spectra the Peptide Calibration Standard mixture (Bruker Daltonics) was used. The MS/MS mode for protein identification was consisted with the following setting: ion source 1, 7.50 kV; ion source 2, 6.75 kV; lens, 3.50 kV; reflectron 1, 29.50 kV; reflectron 2, 14.00 kV; lift 1, 19.00 kV; lift 2, 3.00 kV, pulsed ion extraction time, 80 ns. The control of the instrument, data acquisition, processing and evaluation was performed using the following software platforms: FlexControl 3.4, FlexAnalysis 3.4 and BioTools 3.2 (Bruker Daltonics) The tandem mass spectra were analyzed by searching the SwissProt database with Mascot 2.4.1 search engine (Matrix Science, London, UK). The searches were taxonomically restricted to “*Homo sapiens*”. The general search parameters for the MALDI-TOF data were as follows: no enzyme, peptide precursor mass tolerance: 50 ppm; fragment mass tolerance: 0.7 Da; peptide charge: +1; monoisotopic mass.

### 3.7. Statistical Analysis

In order to examine variables: age, BMI, gestational age at sampling, gestational age at delivery, and birth weight, the Shapiro–Wilk test of normality was used in the first step. For variables with normal distribution, the Levene’s test and the Brown–Forsythe test were subsequently used to examine the equality of variances for the study and control group, followed by the *t*-test to examine the difference between these two groups. For variables, which populations were not normally distributed, the Mann–Whitney *U* test was used for comparison between the study and control group.

Being aware of the small sample size, we have applied different chemometric approaches to find and depict the correlations between the data obtained. For metabolomic and proteomic data analysis both univariate and multivariate statistical analyses were applied. It was indicated that the Mann–Whitney *U* test and *t*-test were the most robust to overcome of descending sample size effect [31]. The variables with *p*-value ≤0.05 in the univariate analyses (*t*-test and the Mann–Whitney *U* test) were found as potentially important features of GDM. The multivariate analyses included Partial Least Squares Discriminant Analysis (PLS-DA) and the Discriminant Function Analysis (DFA). PLS-DA was used for classification of the samples and to get a ranking of studied endogenous compounds contributing to classification of patients between two analyzed groups. PLS-DA models were validated by permutation tests [32]. DFA was applied in order to assess the classification capabilities of a group of selected peptides and amino acids, including sensitivity and specificity. Prior to multivariate analyses the normalization by sum, as well as the transformation by taking the natural logarithm and autoscaling of the input data were applied.

The statistical analyses were performed using the STATISTICA 10.0 (Statsoft Polska: Cracow, Poland) and MetaboAnalyst 3.0 web portal [33]. In all statistical analyses a *p*-value ≤0.05 was considered statistically significant.

## Supplementary Materials

Supplementary materials can be found at http://www.mdpi.com/1422-0067/16/12/26133/s1.
