# Supplementary material for: A Combined Metabolomic and Proteomic Analysis of Gestational Diabetes Mellitus
Source: Int J Mol Sci. 2015 Dec 16;16(12):30034–45. doi: 10.3390/ijms161226133 (PMC4691080; doi:10.3390/ijms161226133)
Supplement: Supplementary file 1 [file ijms-16-26133-s001.pdf]

Supplementary Materials: A Combined Metabolomic and Proteomic Analysis of Gestational Diabetes Mellitus

Joanna Hajduk, Agnieszka Klupczyńska, Paweł Dereziński, Jan Matysiak, Piotr Kokot, Dorota Nowak, Marzena Gajęcka, Ewa Nowak-Markwitz and Zenon J. Kokot

Table S1. The concentrations of determined free amino acids using the LC-ESI-QqQ-MS/MS method in plasma of the study and control group. Bold font indicates *p*-values lower than 0.05.

Amino Acid	Abbreviation	Calculated Concentration in the Samples (μM)								*p*-Value *
		Study Group (*n* = 18)				Control Group (*n* = 13)				
		Average	Median	Min.	Max.	Average	Median	Min.	Max.	
Taurine	Tau	23.8	24.2	17.7	31.1	26.5	23.9	18.6	40.5	0.181
L-Asparagine	Asn	38.7	37.1	30.1	55.5	47.5	48.9	32.6	57.1	**0.001**
L-Serine	Ser	102.3	103.6	78.6	132.6	94.9	95.9	62.5	126.1	0.258
Glycine	Gly	164.4	164.0	122.1	251.9	172.2	173.9	127.5	217.5	0.476
Hydroxy-L-proline	Hyp	9.7	9.8	6.2	15.0	11.3	9.3	6.3	26.3	0.645
Ethanolamine	EtN	5.1	5.1	4.4	6.1	4.6	4.6	3.2	6.8	0.054
L-Glutamine	Gln	278.2	282.8	228.9	346.5	303.7	318.7	234.5	401.7	0.103
L-Asparticacid	Asp	6.5	6.4	4.5	9.5	6.0	5.6	3.6	10.9	0.180
L-Citrulline	Cit	15.3	15.1	10.0	20.5	12.5	12.2	8.3	19.5	**0.015**
L-Threonine	Thr	154.4	152.8	117.4	197.6	161.5	168.6	108.8	225.0	0.547
Sarcosine	Sar	1.6	1.7	1.3	2.0	1.7	1.7	1.6	2.0	0.246
β-Alanine	bAla	28.5	26.0	19.7	46.5	28.4	27.9	18.5	40.5	0.889
L-Alanine	Ala	298.2	297.5	22.1	457.4	341.3	348.9	211.5	423.9	0.148
L-Glutamicacid	Glu	48.9	48.8	25.6	74.6	42.0	35.6	26.6	65.1	0.160
L-Histidine	His	68.8	66.4	57.7	98.0	68.5	67.2	57.8	79.3	0.704
1-Methyl-L-histidine	1MHis	1.9	1.1	0.2	8.4	2.0	1.5	0.2	9.2	0.734
3-Methyl-L-histidine	3MHis	1.5	1.5	1.0	2.1	1.7	1.8	1.2	2.4	0.124
L-α-Aminoadipicacid	Aad	0.6	0.6	0.3	1.1	0.7	0.6	0.5	1.0	0.246
γ-Amino-*n*-butyric acid	GABA	0.1	0.1	0.1	0.3	0.2	0.2	0.1	0.2	0.589
D. L-β-Aminoisobutyric acid	bAib	1.5	1.4	0.7	3.3	1.5	1.4	0.8	2.4	0.825
L-α-Amino-*n*-butyric acid	Abu	13.8	14.6	6.1	24.9	11.8	10.1	5.8	30.4	0.207
L-Arginine	Arg	39.5	39.8	18.4	53.2	34.0	32.7	19.6	46.8	0.103

Table S1. *Cont.*

Amino Acid	Abbreviation	Calculated Concentration in the Samples (μM)								*p*-Value *
		Study Group (n = 18)				Control Group (n = 13)				
		Average	Median	Min.	Max.	Average	Median	Min.	Max.	
L-Proline	Pro	148.3	144.2	102.4	216.5	146.1	152.8	97.2	175.2	0.860
L-Ornithine	Orn	30.6	29.6	14.4	44.3	37.0	36.7	24.9	53.4	**0.038**
L-Cystine	Cys	1.2	1.2	0.4	1.2	1.2	1.2	0.5	2.0	0.562
δ-Hydroxylysine	Hyl	2.2	2.2	1.5	3.1	2.2	2.2	1.5	3.0	1.000
L-Lysine	Lys	120.3	110.3	77.6	190.3	132.6	125.2	109.4	180.9	0.052
L-Methionine	Met	18.8	18.0	15.0	25.9	20.5	20.1	13.2	33.6	0.262
L-Valine	Val	163.3	157.4	125.0	230.6	183.2	182.3	118.0	246.3	**0.029**
L-Tyrosine	Tyr	31.5	30.3	21.6	45.4	32.5	33.6	23.0	43.5	0.671
L-Isoleucine	Ile	48.2	48.6	32.1	77.1	50.3	49.0	38.4	66.7	0.578
L-Leucine	Leu	73.8	72.8	51.3	113.5	77.5	75.7	54.1	99.2	0.512
L-Phenylanalanine	Phe	43.2	42.3	37.0	59.1	42.0	42.4	32.7	50.2	0.968
L-Tryptophan	Trp	36.6	36.0	29.0	46.4	36.7	37.4	26.9	45.6	0.948

* *p* values were calculated according to *t*-test or Mann-Whitney *U* test.

Table S2. The intensities of peptide ions acquired using the MALDI-TOF-MS method in plasma of the study and control group. Bold font indicates *p*-values lower than 0.05.

Peptide Ion (*m*/*z*)	Intensities in the Samples								*p*-Value *
	Study Group (*n* = 18)				Control Group (*n* = 13)				
	Average	Median	Min.	Max.	Average	Median	Min.	Max.	
1005.07	16.1	15.6	4.8	27.3	17.5	17.4	5.4	36.9	0.565
1020.82	45.3	41.2	16.6	84.4	49.2	52.1	32.0	71.9	0.559
1030.73	13.1	13.8	4.4	27.0	16.4	14.2	6.3	38.8	0.222
1037.09	58.0	48.0	30.9	108.1	57.7	58.0	12.7	97.3	0.795
1044.23	34.6	35.4	2.5	55.0	42.8	41.7	12.0	58.9	0.070
1053.01	17.5	14.0	1.0	51.2	23.1	18.8	8.0	68.0	0.289
1060.99	606.6	637.2	79.0	778.0	660.3	669.9	390.2	776.4	0.082
1077.09	170.4	175.1	11.0	349.5	228.9	230.6	48.2	352.2	0.115
1082.84	117.0	121.5	32.1	216.3	112.8	102.5	80.1	159.0	0.752
1093.29	17.7	17.1	10.3	24.9	18.8	17.8	13.8	31.2	0.479
1099.17	314.9	313.2	27.7	496.0	299.0	284.2	101.8	525.9	0.702
1109.49	18.0	17.5	1.7	40.1	20.9	21.9	1.0	45.9	0.535
1114.48	12.5	11.1	0.0	40.5	16.3	7.8	4.3	54.9	0.617
1121.27	53.8	53.8	3.3	100.5	43.4	36.9	15.9	92.8	0.247
1125.18	64.7	71.0	24.1	95.6	78.6	70.4	57.7	165.6	0.368
1131.10	16.8	12.1	0.0	55.2	17.3	10.6	0.0	67.6	0.617
1137.68	31.2	25.0	9.8	73.0	31.4	28.9	14.3	61.7	0.795
1153.72	27.0	23.9	14.3	46.0	28.4	30.5	8.9	48.1	0.719
1163.01	17.8	18.8	8.8	24.0	18.4	19.1	13.3	26.0	0.742
1169.55	32.6	27.4	5.9	57.8	35.0	37.1	4.5	54.1	0.678
1181.45	12.2	11.0	6.1	18.3	12.1	12.2	4.7	20.7	0.949
1193.26	57.3	52.9	4.9	128.5	71.2	78.4	32.9	102.2	0.216
1212.70	72.5	73.5	17.8	129.0	82.6	73.2	48.5	143.8	0.327
1226.06	13.7	13.0	6.4	21.4	16.5	16.4	9.4	22.6	0.070
1234.75	26.6	28.2	3.6	61.6	24.0	21.6	13.5	37.2	0.526
1241.61	11.2	10.9	0.6	22.0	11.5	12.7	0.2	26.2	0.900
1250.00	19.3	22.0	4.2	28.1	22.4	23.3	10.3	32.6	0.307
1257.86	20.6	22.1	5.5	39.9	18.7	19.6	1.1	31.9	0.567
1264.51	20.0	11.4	1.5	100.3	13.0	9.9	2.9	30.8	0.674
1269.60	17.0	10.2	3.3	82.4	20.2	15.2	6.3	43.1	0.105
1278.25	17.6	14.9	0.0	89.4	8.7	9.5	0.0	21.5	0.254
1286.21	17.5	16.3	5.7	35.0	17.7	17.5	10.4	25.9	0.925
1301.65	10.7	8.8	2.9	24.6	10.6	12.1	2.7	19.0	0.857
1343.62	15.7	14.9	0.0	55.4	13.8	12.4	7.2	22.1	0.674
1350.81	21.9	15.8	4.1	79.3	13.5	14.9	5.9	27.2	0.254
1363.94	14.4	10.5	1.7	29.6	16.0	18.9	5.3	28.4	0.614
1370.10	39.5	42.9	1.2	72.3	26.7	27.5	7.6	51.7	**0.026**
1380.63	15.5	13.0	0.6	36.8	21.2	16.1	3.8	40.5	0.200
1385.60	23.1	22.6	2.3	45.6	24.8	25.1	13.3	36.7	0.673
1419.58	31.4	32.5	2.3	55.8	21.7	19.7	7.2	33.5	**0.032**
1425.22	11.7	10.3	1.0	44.7	16.0	14.6	7.9	33.5	0.052
1435.03	16.9	15.0	1.7	43.4	11.7	11.8	7.7	15.7	**0.048**
1450.82	51.8	57.0	4.3	86.5	63.4	68.7	34.0	93.0	0.289
1466.64	129.9	98.9	6.5	402.4	73.9	60.8	40.2	170.6	0.075
1482.32	15.7	14.8	0.0	53.6	17.0	16.2	6.1	33.1	0.617
1488.59	19.7	13.3	2.2	74.4	7.0	6.5	3.1	10.8	**0.006**
1499.94	141.4	151.3	13.4	256.7	181.4	182.6	104.8	256.2	0.124
1521.57	34.3	32.4	2.5	86.3	31.2	31.3	13.4	55.2	0.635
1537.77	152.5	155.4	5.5	382.4	116.6	111.3	62.8	200.9	0.154
1553.87	91.0	82.9	3.8	188.5	98.0	103.3	37.7	171.6	0.649
1575.86	39.4	41.4	0.8	60.5	36.1	35.3	22.6	58.6	0.533
1607.09	47.7	43.4	3.9	91.9	45.7	46.0	15.8	77.1	0.813

Table S2. *Cont.*

Peptide Ion (m/z)	Intensities in the Samples								p-Value *
	Study Group (n = 18)				Control Group (n = 13)				
	Average	Median	Min.	Max.	Average	Median	Min.	Max.	
1617.69	86.6	85.8	2.0	239.0	60.4	49.0	26.9	123.8	0.222
1640.30	31.2	25.5	2.4	161.6	25.6	21.9	7.8	67.8	0.764
1720.22	15.2	11.3	2.6	40.3	12.4	12.3	7.1	19.0	0.920
1748.12	128.6	141.1	8.8	240.9	133.8	144.2	69.6	178.6	0.793
1754.88	37.6	42.6	3.8	67.4	47.3	49.9	21.5	62.7	0.105
1770.06	18.1	12.5	0.5	55.8	13.6	14.1	1.6	26.6	0.826
1777.62	38.6	33.9	1.0	86.0	29.5	29.6	15.5	47.3	0.119
1790.40	23.9	28.1	0.8	52.8	24.2	24.6	4.6	38.6	0.950
1831.02	12.7	13.2	0.7	21.6	11.8	11.4	5.4	23.8	0.670
1848.29	61.8	58.2	3.5	109.2	57.6	53.8	30.8	84.1	0.607
1866.56	28.7	23.7	3.3	71.1	24.3	25.0	11.4	37.1	0.376
1897.55	132.7	139.4	12.5	223.6	144.8	142.3	62.2	209.1	0.496
1919.57	13.9	14.2	0.0	34.2	10.5	11.4	1.5	19.6	0.209
1928.59	32.8	30.4	1.8	69.3	36.8	31.2	20.4	66.9	0.572
1935.57	35.4	29.4	3.5	68.9	34.2	35.5	21.6	50.8	0.562
1945.86	65.8	65.2	3.0	173.7	69.4	74.3	18.7	99.3	0.816
1952.26	30.3	35.8	0.0	77.7	21.1	18.2	1.2	41.7	0.589
1967.87	10.7	7.7	0.1	39.5	9.2	10.8	0.6	13.1	0.920
1981.97	11.0	5.5	0.3	57.8	9.5	8.7	1.5	20.1	0.347
1993.69	21.2	16.9	1.8	53.0	12.4	11.2	5.3	30.9	0.057
2022.64	172.5	164.4	15.1	306.5	190.6	165.4	88.6	297.9	0.418
2034.71	19.8	19.8	0.0	52.3	13.2	13.3	1.0	28.7	0.160
2044.64	25.4	21.8	1.1	65.0	22.6	21.8	9.8	46.0	0.535
2061.79	17.3	15.5	2.0	41.8	13.0	12.4	5.2	22.9	0.154
2082.90	61.0	67.6	5.2	118.6	69.1	72.2	24.8	100.4	0.435
2108.63	49.1	48.7	2.5	119.6	49.9	42.6	20.6	99.5	0.947
2116.69	32.6	34.7	0.6	89.4	30.2	32.1	8.5	50.9	0.746
2126.66	13.3	14.9	0.2	30.8	17.2	16.8	7.2	24.7	0.170
2168.63	50.4	60.7	5.3	85.8	70.6	74.5	31.4	100.9	**0.026**
2190.48	54.3	53.6	2.6	127.2	53.4	51.1	24.0	98.3	0.937
2210.83	51.7	53.0	8.9	84.9	71.4	66.5	41.5	115.6	**0.027**
2229.94	67.3	60.5	4.6	227.0	41.8	40.1	10.2	84.4	0.509
2238.78	20.0	18.3	1.0	62.2	24.3	25.5	6.7	49.7	0.417
2251.32	13.6	9.9	2.0	31.5	8.8	8.3	2.4	14.9	0.327
2272.80	31.9	40.5	2.3	54.7	45.7	44.3	21.9	66.7	0.089
2283.91	29.6	20.4	1.5	81.8	15.8	13.1	5.0	46.7	0.089
2288.31	20.9	19.6	3.9	36.3	17.0	16.4	10.4	30.6	0.124
2311.84	43.0	36.9	3.8	100.4	53.0	56.9	20.8	89.3	0.264
2358.10	82.0	85.7	5.7	166.2	89.9	97.8	33.0	133.9	0.602
2366.74	30.6	24.1	1.9	81.8	45.6	50.5	10.1	74.9	0.073
2410.09	13.6	15.7	0.2	27.5	11.5	10.9	1.8	27.3	0.435
2425.62	19.4	14.3	1.3	41.2	23.6	23.3	11.2	44.5	0.314
2474.70	14.4	15.4	0.6	38.2	15.1	15.2	9.3	22.8	0.795
2485.95	68.5	62.9	4.0	153.0	86.8	84.8	38.7	144.9	0.162
2547.25	46.5	52.1	1.1	120.5	47.1	43.5	20.3	83.0	0.963
2553.54	70.3	70.3	5.6	136.4	82.2	82.6	46.7	116.7	0.325
2567.45	23.1	18.7	0.9	50.2	25.5	23.9	9.4	55.1	0.412
2660.94	57.2	54.3	3.3	148.1	56.1	59.2	16.8	86.4	0.459
2682.92	25.8	24.6	1.3	65.3	29.3	26.6	8.9	60.4	0.545
2726.10	13.7	12.9	1.0	43.0	12.3	11.9	3.1	19.7	0.984
2756.59	54.6	52.3	6.4	114.5	72.5	65.4	31.5	129.6	0.070
2769.99	43.6	40.6	3.9	98.5	40.6	35.7	22.0	61.4	0.685
2863.08	125.9	137.3	8.3	189.4	137.1	142.7	67.0	220.0	0.538

Table S2. *Cont.*

Peptide Ion (*m*/*z*)	Intensities in the Samples								*p*-Value *
	Study Group (*n* = 18)				Control Group (*n* = 13)				
	Average	Median	Min.	Max.	Average	Median	Min.	Max.	
2883.06	41.9	50.4	3.5	71.0	46.0	49.2	16.1	70.2	0.889
2902.32	15.4	15.6	0.8	29.0	13.6	12.9	3.9	21.2	0.499
2913.15	9.6	3.9	0.0	37.5	26.0	25.4	4.4	56.8	**0.002**
2933.15	100.0	99.2	7.2	206.8	92.1	98.5	25.8	146.3	0.678
2946.48	19.8	19.5	1.5	44.4	15.8	15.2	6.2	33.9	0.302
2954.76	14.8	10.6	1.7	45.7	8.7	9.7	0.0	19.4	0.412
3040.35	18.7	17.5	0.2	35.1	19.0	11.2	5.6	61.0	0.589
3061.23	28.0	26.2	5.1	99.4	39.6	35.0	12.3	77.8	0.123
3118.42	13.4	12.7	0.3	34.0	12.8	13.9	3.1	23.9	0.843
3158.72	59.8	63.0	12.6	95.7	83.4	85.5	53.8	114.6	**0.011**
3182.03	9.3	9.7	1.7	19.7	11.2	10.8	4.3	20.5	0.313
3192.53	35.7	36.5	2.8	82.8	35.8	39.1	16.4	50.1	0.674
3241.34	47.1	51.2	4.3	70.5	51.0	55.1	18.3	83.5	0.734
3262.60	15.0	14.1	1.0	30.4	12.0	11.2	6.6	19.4	0.169
3274.46	18.6	18.8	0.2	51.8	17.7	16.8	4.6	31.9	0.866
3363.39	14.6	15.2	0.3	32.6	14.8	15.0	7.9	23.7	0.960
3430.44	28.2	31.0	3.2	60.4	44.9	46.1	29.0	58.6	**0.003**
3521.79	27.2	28.7	2.1	55.4	26.9	30.1	9.2	46.5	0.962
3633.53	16.8	17.8	0.2	43.6	17.4	17.7	8.9	25.7	0.853
3681.87	15.6	17.7	2.8	25.7	19.7	22.7	10.7	26.6	0.123
3772.27	9.8	10.3	1.8	21.9	10.4	11.9	4.5	15.6	0.746
3814.27	22.3	27.9	2.2	41.6	24.2	24.8	9.0	40.3	0.952
3868.05	25.7	27.4	2.6	50.5	26.2	25.7	13.0	43.9	0.927
3955.79	35.3	34.0	5.6	74.9	40.0	44.5	16.6	60.0	0.531
4092.82	25.7	26.1	1.8	52.4	27.4	28.8	5.8	52.3	0.764
4111.89	26.8	27.1	7.9	54.1	39.7	37.2	28.4	52.9	**0.002**
4250.07	12.0	9.7	0.9	26.1	8.5	7.1	2.9	22.9	0.435
4282.12	37.2	33.5	4.0	93.5	40.2	40.1	15.3	70.9	0.327
4298.81	11.4	9.5	1.8	34.0	13.1	11.5	6.4	29.3	0.721
4418.32	66.6	61.8	8.3	149.5	53.5	52.9	25.2	103.5	0.302
4437.76	40.8	40.3	6.1	99.2	54.7	51.9	43.3	71.6	**0.028**
4574.22	77.6	68.9	30.4	132.6	85.9	80.4	53.8	124.6	0.431
4590.58	24.2	23.9	8.8	39.6	32.8	32.7	18.0	44.5	**0.007**
4713.01	12.5	9.4	2.0	43.4	8.6	7.3	4.1	16.5	0.484
5002.29	33.6	32.5	1.0	62.2	29.7	23.1	9.8	52.7	0.554
5159.84	78.2	79.5	4.8	147.4	89.0	84.8	46.8	142.4	0.426
5904.15	14.9	15.4	1.9	30.6	16.0	16.5	4.3	30.2	0.719
6432.43	13.4	11.9	4.9	26.5	16.5	13.6	4.9	32.0	0.225
6630.80	33.8	36.5	14.2	52.0	38.3	40.2	16.1	52.0	0.301
6646.56	14.7	12.3	3.8	31.0	14.5	10.6	2.2	43.0	0.764
8603.13	9.6	8.8	2.1	22.8	12.1	12.8	5.6	19.8	0.210
9134.67	19.4	18.6	0.5	42.4	20.3	20.4	5.0	32.4	0.819
9150.01	19.7	18.1	10.2	40.0	19.7	19.6	9.4	32.0	0.999
9424.76	19.3	17.7	2.6	39.9	19.0	19.8	8.0	29.9	0.927
9440.47	14.5	11.3	5.3	46.3	13.6	13.5	2.0	25.8	0.984

* *p*-values were calculated according to *t*-test or Mann-Whitney *U* test.

Table S3. The Mascot search results of the *m*/*z* 2911.4712; 1465.6566.

Protein ID	Observed (*m*/*z*)	Mr (expt)	Mr (calc)	ppm	Mascot Score	Peptide Sequence
APOA4_HUMAN	2911.4712	2910.4639	2910.4584	1.91	153	R.GNTEGLQKSLAELGGHLDQQVEEFRR.R
FIBA_HUMAN	1465.6566	1464.6494	1464.6481	0.85	140	A.DSGEGDFLAEGGGVR.G

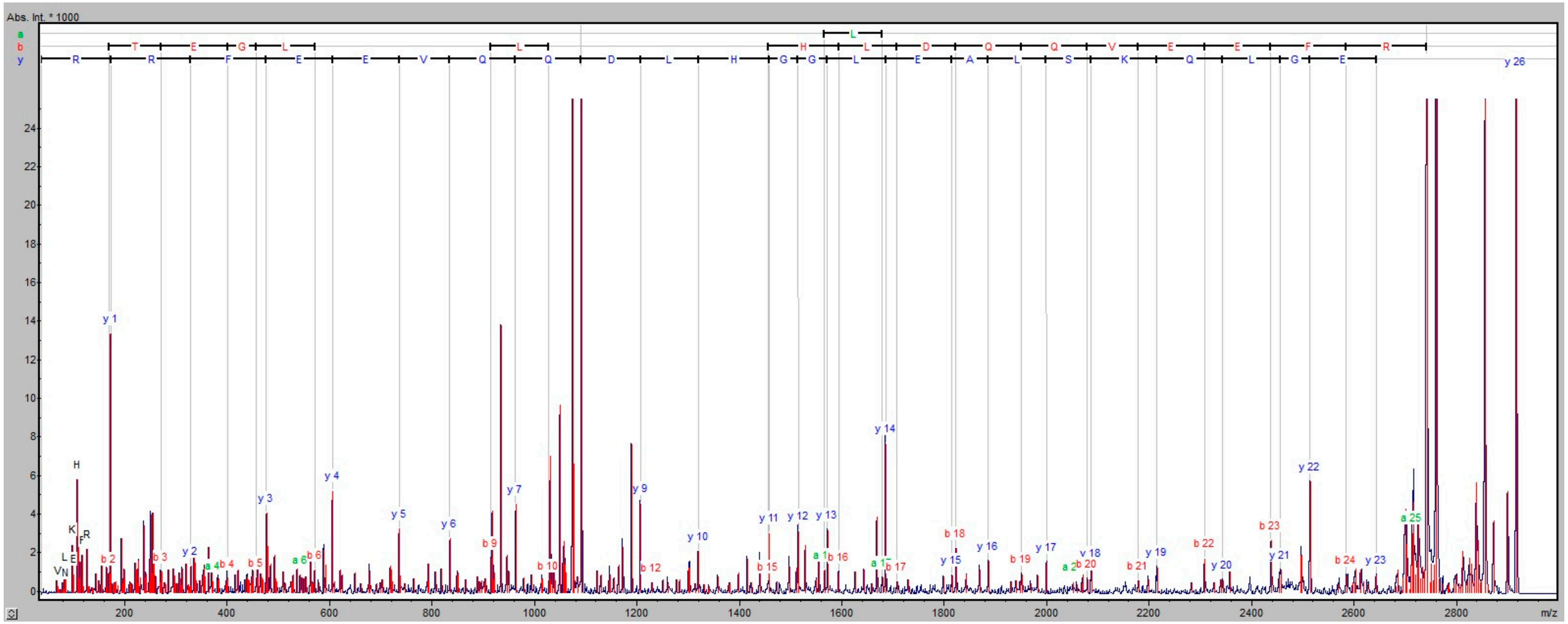

Figure S1. The MS/MS spectrum of *m*/*z* 2911.4712 with annotation of a-, b- and y-ions. Peptide sequence: R.GNTEGLQKSLAELGGHLDQQVEEFRR.R.

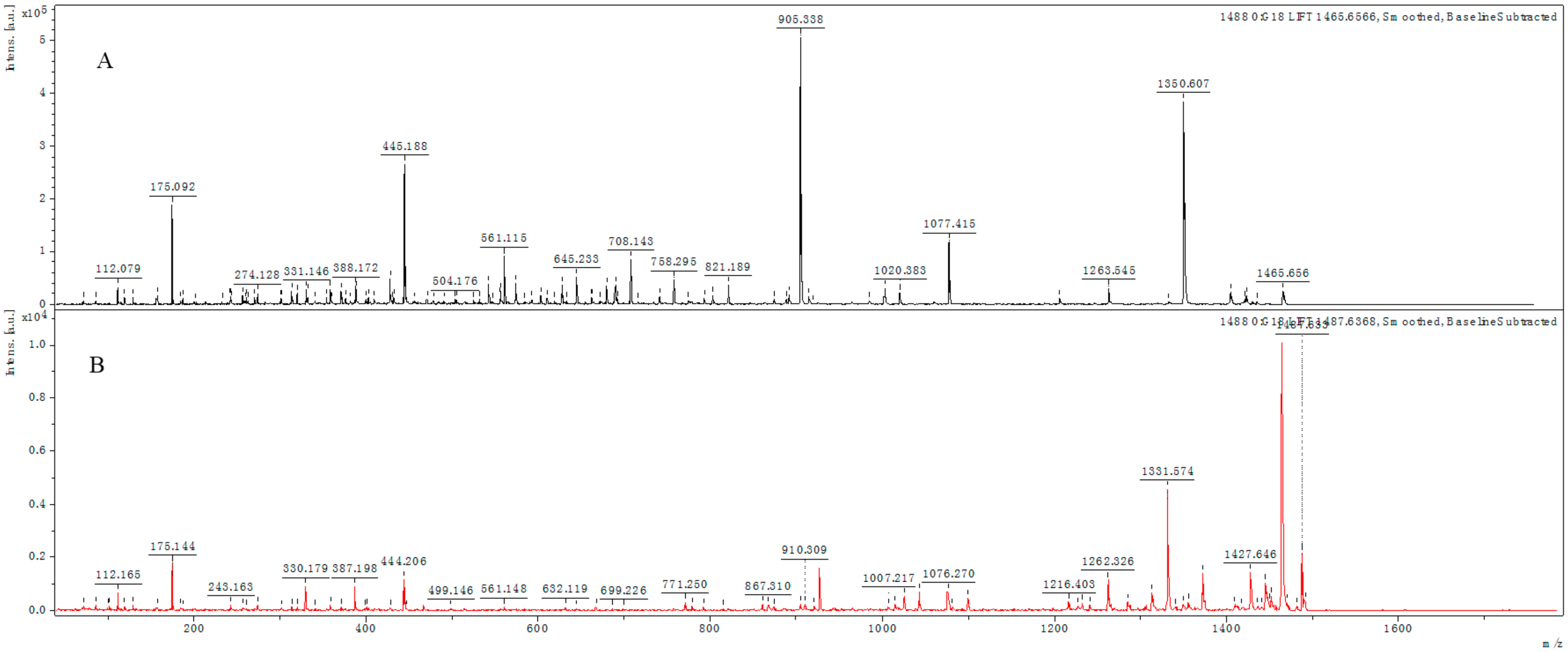

Figure S2. The comparison of MS/MS spectra of *m*/*z* signals 1465.6566 (**A**) *vs*. 1487.6368; (**B**) processed in Flex Analysis 3.4.
